# Suppression of Macrophage Activation by Sodium Danshensu via HIF-1α/STAT3/NLRP3 Pathway Ameliorated Collagen-Induced Arthritis in Mice

**DOI:** 10.3390/molecules28041551

**Published:** 2023-02-06

**Authors:** Danbin Wu, Jia Xu, Wei Jiao, Lijuan Liu, Jiahui Yu, Mingying Zhang, Guangxing Chen

**Affiliations:** 1 First Clinical Medical School, Guangzhou University of Chinese Medicine, Guangzhou 510405, China; 2 Lingnan Medical Research Center of Guangzhou University of Chinese Medicine, Guangzhou 510405, China

**Keywords:** rheumatoid arthritis, sodium Danshensu, hypoxia-inducible factor-1α, signal transducer and activator of transcription 3, NOD-like receptor protein 3 inflammasome

## Abstract

It is still a clinical challenge to sustain the remission of rheumatoid arthritis (RA); thus, identifying more effective and safer agents for RA treatment remains an urgent demand. We investigated the anti-arthritic activity and potential mechanism of action of sodium Danshensu (SDSS), a structurally representative water-soluble derivative of Danshen, on collagen-induced arthritis (CIA) mice. Our results showed that paw edema, synovium hyperplasia, bone destruction, and the serum levels of both IL-1β and IL-6 were ameliorated by SDSS (40 mg/kg·d) in CIA mice. In addition, there was no difference between SDSS and methotrexate (MTX, 2 mg/kg·3d) treatment in the above indicators. Further mechanism studies illustrated that SDSS inhibited IL-1β secretion by downregulating the HIF-1α/STAT3/NLRP3 pathway in macrophages. On the other hand, HIF-1α accumulation and HIF-1α/STAT3/NLRP3 pathway activation by IOX4 stimulation reduced the therapeutic effect of SDSS. These findings demonstrate that SDSS displays anti-arthritic activity in CIA mice and prevents proinflammatory cytokines secretion in macrophages by suppressing the HIF-1α/STAT3/NLRP3 pathway.

## 1. Introduction

Rheumatoid arthritis (RA) is an autoimmune disease characterized by persistent synovitis, which results in joints destruction and even disability [1]. Although the mechanism underlying persistent synovitis remains elusive, extensive studies have identified that the increasing infiltration of macrophages into the synovium is an early hallmark of the active synovitis [2]. These macrophages are considered to be the central effectors of synovitis and produce a large number of interleukin (IL)-1β, IL-6 and tumor necrosis factor (TNF)-α [3]. Macrophages can be activated by a range of pathogen-associated molecular patterns (PAMPs), damage-associated molecular patterns (DAMPs) and proinflammatory cytokines. Recent evidence has revealed that metabolism and the immunological state of macrophages are inextricably linked [4]. For the maintenance of energy homeostasis, activated macrophages shift glucose metabolism from oxidative to glycolytic [5]. This shifting results in the accumulation of metabolic intermediates in macrophages, which in turn act as signaling molecules to aggravate the inflammatory response [6]. Accordingly, the reprogramming of immunometabolism in macrophages could potentially be a new approach to RA treatment.

Hypoxia-inducible factor (HIF)-1α plays a pivotal role in the hypoxia adaptation response [7]. A decrease in synovial oxygen tension as well as an increase in HIF-1α levels have been observed in RA synovium. The accumulation of HIF-1α leads to a shifting of glucose metabolism and increases proinflammatory cytokine release in macrophages. Further research has revealed that HIF-1α aggravated the expression and phosphorylation of signal transducer and activator of transcription (STAT) 3 [8]. As a transcriptional regulator, STAT3 has essential roles in the immune response [9]. The activation of STAT3 (expression and phosphorylation) is associated with the progress of RA; clinically effective therapies consistently reduce the activation of STAT3 [10,11]. Available data suggest that the phosphorylated STAT3 (p-STAT3) is translocated into the nucleus and is bound to the NOD-like receptor protein (NLRP) 3 promoter region, as well as facilitates acetylation of histone H3 and H4 on the NLRP3 promoter, thereby aggravating NLRP3 expression and NLRP3 inflammasome activation [12,13]. The NLRP3 inflammasome is a cytoplasmic multimolecular platform for IL-1β maturation and is essential for inflammatory activation of macrophages [14,15,16]. However, aberrant NLRP3 inflammasome activation has been linked with immune-mediated pathology, including RA [17]. In summary, the HIF-1α/STAT3/NLRP3 axis might connect the metabolism reprogramming and inflammatory activation of macrophages under hypoxia, and we hypothesize that it could potentially be a remedial target for RA.

Danshen, the dried root or rhizome of *Salvia miltiorrhiza* Bge., is a traditional Chinese medicine for promoting blood circulation and restoring/enhancing immunity [18]. Today, it is frequently utilized in decoctions for RA treatment. Danshensu (DSS) is a structurally representative water-soluble, phenolic compound of Danshen. Recent studies have demonstrated that DSS ameliorates inflammatory cytokines secretion and improves histopathological changes in mice with acute lung inflammation by inhibiting the TLR4/NF-κB p65 pathway [19]. In addition, a novel compound derived from DSS, [4-(2-acetoxy-3-((R)-3-(benzylthio)-1-methoxy-1-oxopropan-2-ylamino)-3-oxopropyl)-1,2-phenylene diacetate], exhibits a colitis-protecting effect by modulating NLRP3 inflammasome activation [20]. Sodium Danshensu (SDSS) or salvianic acid A sodium is the sodium salt form of DSS, which is more stable in nature. SDSS also possesses a variety of pharmacological actions, such as antineoplastic [21], anti-oxidative [22] and anti-inflammatory activity [23]. Considering these findings, we wonder if SDSS exerts anti-arthritis properties by inhibiting HIF-1α/STAT3/NLRP3 axis.

The therapeutic effect of SDSS in CIA was detected in the present experiment. We describe how SDSS is a potent HIF-1α/STAT3/NLRP3 pathway inhibitor that has been found to be active in CIA mice models (in vivo) and in inflammatory macrophages models (in vitro).

## 2. Results

### 2.1. SDSS Improves Body Weight and Splenic and Hepatosomatic Index

**Figure 1:**
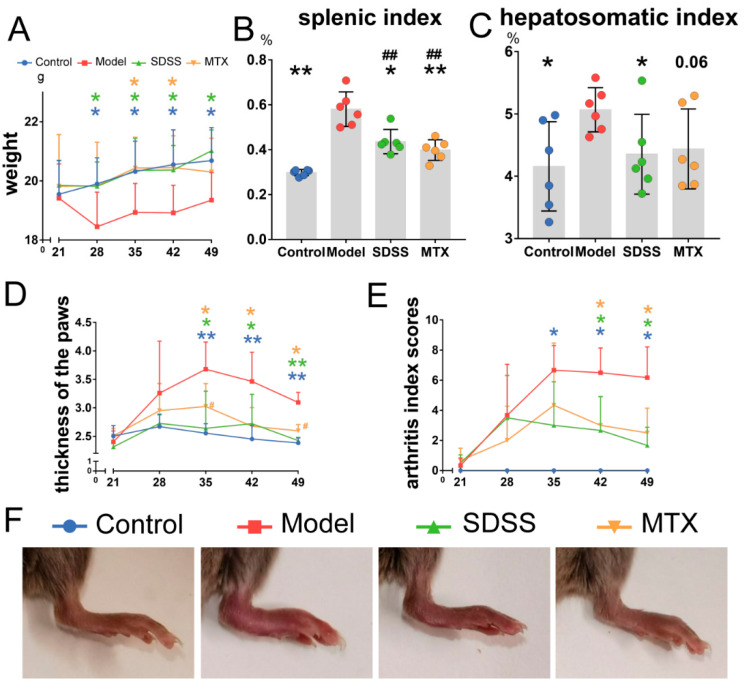
SDSS improves body weight, hepatosomatic index, splenic index and joint swelling in CIA mice. (**A**) Body weight was recorded weekly during the experiment. (**B**) Statistical analysis of splenic index (%, spleen weight/body weight × 100). (**C**) Statistical analysis of hepatosomatic index (%, liver weight/body weight × 100), *p* = 0.06 vs. the model group. (**D**) The thickness of hind paws was measured weekly with digital calipers. (**E**) The clinical arthritis index scores were determined weekly according to a scoring system of 0–4 for each limb. (**F**) Representative paw images of mice in different treatment groups. Data are presented as the mean ± SD. * *p* < 0.05, ** *p* < 0.001 vs. the model group. ^##^ *p* < 0.001 vs. the control group.

Body weight steadily increased in mice of the control group throughout the experiment (Figure 1A). There was a weight decrease in the model group on day 28 (one week after the booster immunization). As a result, these mice were much lighter than those in the control group after day 28. However, the weights of the SDSS and methotrexate (MTX) groups increased smoothly throughout the experiment, and no differences were detected when compared with the control group. In addition, when compared to the model group, mice in the SDSS and MTX groups were much heavier from days 28 to 49 and from days 35 to 42, respectively.

Figure 1B,C shows that the splenic index and hepatosomatic index in the model group were greatly increased. The splenic indexes of the SDSS and MTX groups were much lower than that of the model group; however, both of these were still much higher than in the control group. In addition, the hepatosomatic index in the SDSS group was also remarkably decreased when compared to the model group. In the MTX group, a slight decrease with no statistical difference (*p* = 0.06) in the hepatosomatic index was observed when compared with the model group. Furthermore, no differences were identified between the control, SDSS and MTX groups.

### 2.2. SDSS Ameliorates Joints Swelling and Bone Damage

The induction of CIA was successful; as shown by Figure 1F, visible arthritis development appeared in mice of the model group, and the thickness of paws and the arthritis index scores increased rapidly after the booster immunization, peaking on day 35 (Figure 1D,E). Furthermore, from day 35 to day 49, the model group showed a more extensive elevation in paw thickness and arthritis index score than the control group. The thickness of the paws and the arthritis index scores showed a mild increase after the booster immunization in both the SDSS and MTX groups. In addition, both of these two indicators exhibited a significant decline from days 35 to 49 and from days 42 to 49, respectively, compared to those in the model group.

**Figure 2:**
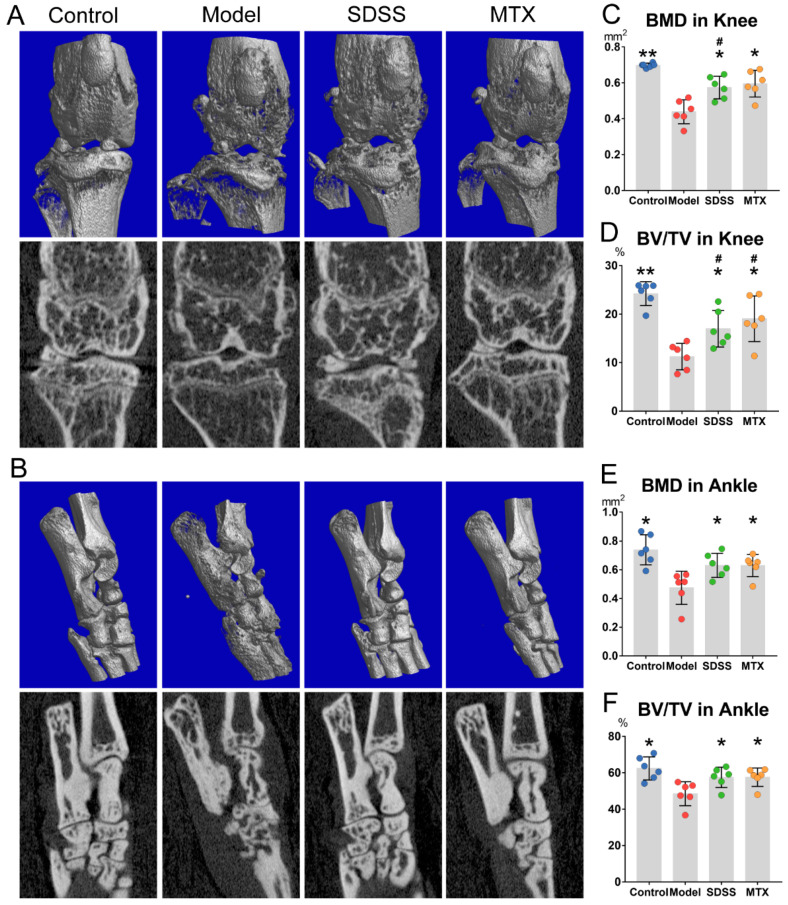
SDSS ameliorates bone damage in CIA mice. (**A**,**B**) Representative reconstructed two-dimensional images (upper panels) and three-dimensional images (lower panels) of knee and ankle joints. (**C**–**F**) Morphometric data of BMD (mm^2^) and BV/TV (%) in knee and ankle joints. Data are presented as the mean ± SD. * *p* < 0.05, ** *p* < 0.001 vs. the model group. ^#^ *p* < 0.05 vs. the control group.

Two- and three-dimensional images of knee and ankle joints are shown in Figure 2A,B. Mice in the model group had an extensive decline in bone volume, as the bone mineral density (BMD) and bone volume/tissue volume (BV/TV) of knees and ankles in these mice were much lower than in the control group (Figure 2C–F). However, both the SDSS and MTX groups showed a significant increase in the BMD and BV/TV of knees compared to the model group, although they were still much lower than in the control group (Figure 2C,D). Moreover, both the SDSS and MTX groups revealed a substantial improvement in the BMD and BV/TV of ankles compared to the model group, and no differences were observed when compared with the control group (Figure 2E,F).

### 2.3. SDSS Reduces Synovium Hyperplasia, as Well as Serum IL-1β and IL-6 Levels

**Figure 3:**
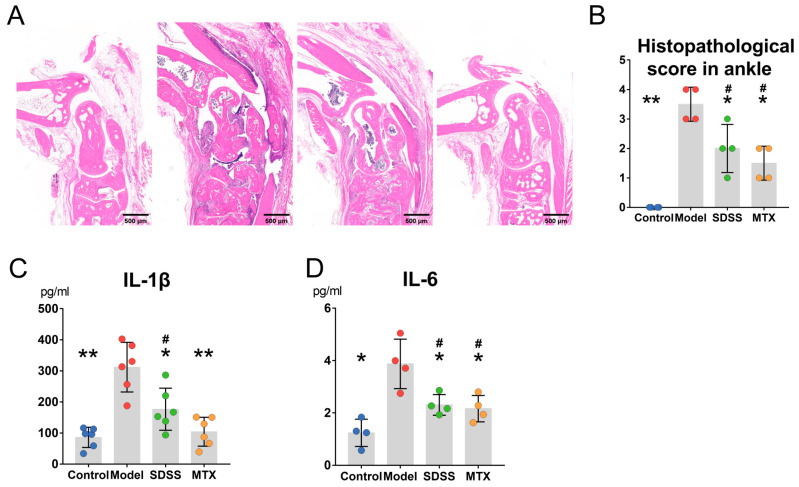
SDSS inhibits inflammation in CIA mice. (**A**) Representative images of HE-stained paraffin sections in ankle joints. (**B**) Semiquantitative score of synovial inflammation in ankle joints. (**C**,**D**) Serum levels of IL-1β and IL-6 were determined by ELISA kits. Data are presented as the mean ± SD. * *p* < 0.05, ** *p* < 0.001 vs. the model group. ^#^ *p* < 0.05 vs. the control group.

Figure 3A shows significant synovium hyperplasia, pannus formation and inflammatory cell infiltration in the ankles of mice with CIA. However, in comparison with the model group, the synovium hyperplasia was significantly relieved in both the SDSS and MTX groups, and the histopathological score was much lower in these two groups (Figure 3B).

As shown by Figure 3C,D, serum IL-1β and IL-6 levels in the model group were considerably elevated. The SDSS group demonstrated lower serum IL-1β and IL-6 levels than the model group, although these were still substantially higher than in the control group. In addition, the MTX group revealed a substantial decline in serum IL-1β and IL-6 levels, compared to the model group. No difference in serum IL-1β levels was observed between the MTX and control groups, whereas the serum IL-6 levels in the MTX group were higher than those in the control group. Furthermore, no differences in the levels of these two cytokines were observed between the SDSS and MTX groups.

### 2.4. SDSS Suppresses HIF-1α, STAT3 and NLRP3 Expression in Peripheral Leukocytes and Synovial Tissue

**Figure 4:**
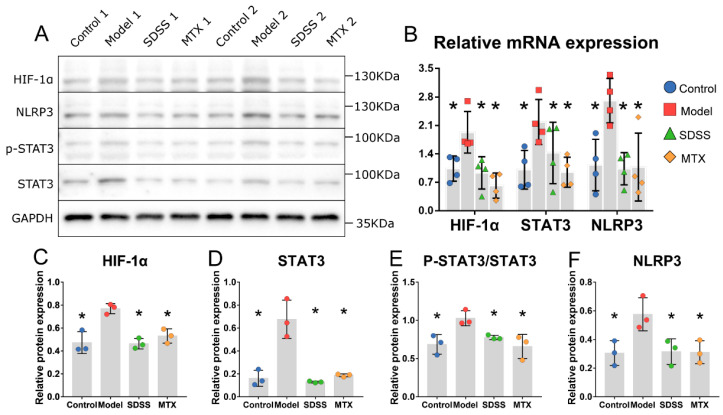
SDSS suppresses the HIF-1α/STAT3/NLRP3 pathway in CIA mice. (**A**) The relative protein expression of HIF-1α, STAT3 and NLRP3 in synovial tissue was determined by Western blotting. (**B**) The relative gene expression of HIF-1α, STAT3 and NLRP3 in peripheral leukocytes was determined by real-time PCR. (**C**–**F**) Statistical analysis of HIF-1α, STAT3, p-STAT3 and NLRP3 protein expression. Data are presented as the mean ± SD. * *p* < 0.05 vs. the model group.

HIF-1α, STAT3, p-STAT3 and NLRP3 protein expression in the synovial tissue of mice in the model group was substantially elevated (Figure 4A). Additionally, HIF-1α, STAT3, p-STAT3 and NLRP3 protein expression in the SDSS and MTX groups was much lower than that in the model group (Figure 4C–F). Among the control and SDSS and MTX groups, no difference in the expression of these four proteins was identified.

Because there was insufficient synovial tissue for RNA extraction, we used peripheral leukocytes as a substitute. In peripheral leukocytes from the model group, HIF-1α, STAT3 and NLRP3 gene transcriptional levels were greatly elevated (Figure 4B). Both the SDSS and MTX groups revealed substantial reductions in HIF-1α, STAT3 and NLRP3 gene transcription compared to the model group. Whereas, among the control and SDSS and MTX groups, no difference in these gene transcriptional levels was observed.

### 2.5. Cytotoxicity Effect of SDSS

**Figure 5:**
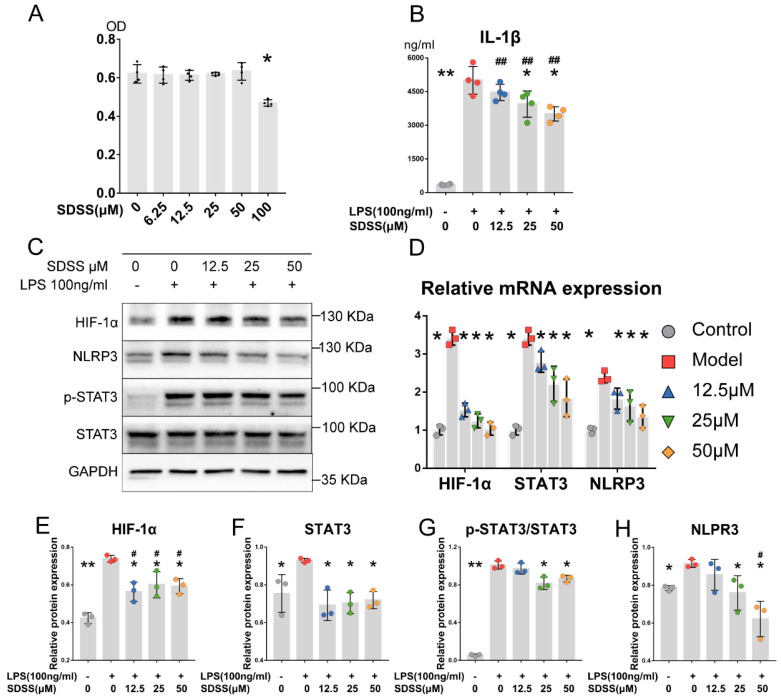
SDSS suppresses the HIF-1α/STAT3/NLRP3 pathway in THP-1-derived macrophages with LPS stimulation. (**A**) THP-1-derived macrophages were treated with or without varying concentrations of SDSS for 24 h, and then, the cell viability was determined by CCK-8 assay (* *p* < 0.05 vs. cell without SDSS treatment). (**B**) THP-1-derived macrophages were pretreated with or without SDSS for 2 h, followed by LPS stimulation for 24 h. IL-1β secretion in cell supernatant was investigated using an ELISA kit. (**C**,**D**) Western blotting and real-time PCR analysis were used to determine the relative expression of HIF-1α, STAT3 and NLRP3 in THP-1-derived macrophages, which were pretreated with or without SDSS for 2 h, followed by LPS stimulation for 24 h. (**E**–**H**) Statistical analysis of HIF-1α, STAT3, p-STAT3 and NLRP3 protein expression. Data are presented as the mean ± SD. * *p* < 0.05, ** *p* < 0.001 vs. the model group. ^#^ *p* < 0.05, ^##^ *p* < 0.001 vs. the control group.

As shown in Figure 5A, the cytotoxicity of SDSS (6.25–100 μM) in macrophages was investigated. No obvious cytotoxicity at doses of SDSS up to 50 μM was observed. Consequently, three lower doses of SDSS, 12.5, 25 and 50 μM, were used in the following studies.

### 2.6. SDSS Reduces LPS-Induced IL-1β Secretion in Macrophages

Different concentrations of SDSS (12.5, 25, and 50 μM) were used to pretreat cells for 2 h before exposure to LPS (100 ng/mL) stimulation for another 24 h. As shown in Figure 5B, IL-1β secretion was dramatically increased by LPS stimulation. Meanwhile, 25 and 50 μM SDSS pre-treatment significantly suppressed IL-1β secretion in a dose-dependent manner.

### 2.7. SDSS Downregulates HIF-1α, STAT3 and NLRP3 Expression in LPS-Induced Macrophages

After SDSS pretreatment followed by LPS induction as described above, cell mRNA and proteins were harvested. As shown in Figure 5D, HIF-1α, STAT3 and NLRP3 gene transcriptional levels were significantly elevated by LPS stimulation, whereas pretreatment with SDSS markedly inhibited LPS-induced HIF-1α, STAT3 and NLRP3 transcription in a dose-dependent manner.

Meanwhile, HIF-1α, STAT3, p-STAT3 and NLRP3 protein expression was sharply increased by LPS stimulation (Figure 5C). In addition, in accordance with mRNA expression, SDSS pretreatment at doses of 12.5, 25, and 50 μM significantly inhibited HIF-1α and STAT3 expression under LPS induction (Figure 5E,F). Additionally, LPS-induced p-STAT3 and NLRP3 expression were markedly suppressed by SDSS pre-treatment at doses of 25 and 50 μM (Figure 5G,H). Consequently, 25 μM SDSS was considered to be a minimum efficacious dose and was chosen for the subsequent experiments.

### 2.8. IOX4 Upregulates HIF-1α Expression in Macrophages

**Figure 6:**
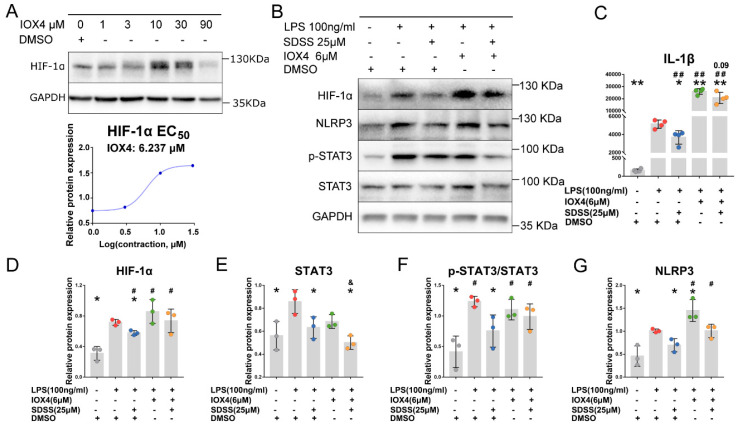
Accumulation of HIF-1α and activation of HIF-1α/STAT3/NLRP3 pathway by IOX4 stimulation abolished the therapeutic effect of SDSS. (**A**) THP-1-derived macrophages were treated with varying concentrations of IOX4 for 24 h. Western blotting was used to determine the relative expression of HIF-1α. The EC_50_ of IOX4 for HIF-1α induction in THP-1-derived macrophages was calculated. (**B**) THP-1-derived macrophages were pretreated with or without SDSS for 2 h, followed by LPS and IOX4 stimulation for 24 h. Western blotting was used to determine the protein expression of HIF-1α, STAT3, p-STAT3 and NLRP3 in THP-1-derived macrophages. (**C**) IL-1β secretion in cell supernatant was investigated using an ELISA kit. *p* = 0.09 vs. the cells stimulated with LPS and IOX4. (**D**–**G**) Statistical analysis of HIF-1α, STAT3, p-STAT3 and NLRP3 protein expression. Data are presented as the mean ± SD. * *p* < 0.05, ** *p* < 0.001 vs. the cells stimulated with LPS alone. ^#^ *p* < 0.05, ^##^ *p* < 0.001 vs. the control group. ^&^ *p*< 0.05 vs. the cells stimulated with LPS and IOX4.

Different concentrations of IOX4 (1, 3, 10, 30 and 90 μM) were used to treat THP-1-derived macrophages for 24 h. As indicated by Figure 6A, IOX4 upregulated HIF-1α expression. The half-maximal effective concentration (EC_50_) for HIF-1α induction in macrophages was 6.237 μM. In order to facilitate calculation and operation, IOX4 at a dose of 6 μM was chosen to conduct the following experiments.

### 2.9. IOX4 Disrupts the Inhibition of SDSS in LPS-Induced HIF-1α, p-STAT3 and NLRP3 Expression in Macrophages

THP-1-derived macrophages were pretreated with or without SDSS (25 μM) for 2 h. Then, these cells were stimulated by LPS (100 ng/mL), together with or without IOX4 (6 μM) for 24 h. IOX4 induction significantly upregulated NLRP3 expression in LPS-induced macrophages (Figure 6B,G). HIF-1α expression was also increased slightly by IOX4 induction, but no significant difference was observed (Figure 6D). IOX4 induction did not change the expression levels of STAT3 and p-STAT3 (Figure 6E,F).

As expected, pretreatment with 25 μM SDSS slightly decreased HIF-1α, p-STAT3 and NLRP3 expression in cells with LPS and IOX4-induced macrophages, and no significant difference was observed (Figure 6D,F,G). However, 25 μM SDSS significantly inhibited STAT3 expression in LPS and IOX4-induced macrophages (Figure 6E).

### 2.10. IOX4 Disrupts the Inhibition of SDSS in LPS-Induced IL-1β Secretion in Macrophages

After the SDSS pretreatment, followed by LPS and IOX4 inducement as described above, the cell supernatant was collected. As shown in Figure 6C, IOX4 induction greatly increased IL-1β secretion in LPS-induced macrophages. Additionally, as expected, 25 μM SDSS pretreatment slightly downregulated IL-1β secretion in cells with LPS and IOX4 stimulation, but the difference was not significant (*p* = 0.09).

## 3. Discussion

RA, known as Bi syndrome in traditional Chinese medicine, has been a disease of great concern to traditional Chinese medicine since ancient times. Many Chinese herbs such as *Tripterygium wilfordii* Hook f., *Paeonia lactiflora* Pall., *Saposhnikovia divaricata* (Turcz.) Schischk. and Danshen have been used to treat Bi syndrome for approximately two millennia. Currently, the efficacy and pharmacological mechanism of these herbs in RA have gained much attention, because there is always a huge demand to identify more effective and safer agents for RA treatment. Meanwhile, with the development of herbal constituent extraction and separation technology, we are able to obtain the purified compounds with clearly defined structures from these herbs, thus facilitating pharmacological research.

SDSS is a structurally representative water-soluble derivative of Danshen and was used to treat CIA mice in the present study. In addition, MTX, a disease-modifying anti-rheumatic drug commonly used in clinical settings, was applied as a positive control drug to evaluate the efficacy of SDSS. This is the first study to demonstrate the anti-arthritis properties of SDSS in CIA mice. Our results show that the paw swelling, arthritis index score and BMD and BV/TV of knee and ankle joints were greatly ameliorated by SDSS treatment, where no differences were detected when compared with MTX treatment. Previous studies have suggested that SDSS is a safe drug with low toxicity, and a dosage of 40 mg/kg·d significantly promoted the healing of ischemia/reperfusion injury in rat [22]. Our results showed that SDSS improved body weight in CIA mice. In addition, SDSS did not show obvious hepatosomatic or splenic toxicity, as it improved the hepatosomatic and splenic index in CIA mice, and no differences were observed when compared with mice that underwent MTX treatment. However, more experiments are needed to investigate the toxicity of the drug, such as whether it affects the functions of the heart, lung, and kidney.

Abundant secretion of proinflammatory cytokines, such as IL-1β and IL-6, along with persistent synovium hyperplasia, is central to the pathogenesis of RA [24]. Our results revealed that SDSS reduced synovial hyperplasia in ankle joints, as well as proinflammatory cytokine levels in serum. Macrophages have been condemned as major participants in synovitis, because of their huge population in the inflamed synovium and powerful secretion of proinflammatory cytokines [2]. The accumulation and activation of macrophages in the synovium correlates to disease activity and has been considered as a sensitive biomarker of the response to therapeutic interventions in RA [25,26]. Consequently, the inhibition of macrophage activation remains a key therapeutic goal for inflammation mitigation in RA treatment.

The RA synovium is characterized by hypoxia and is rich in HIF-1α, a “master regulator” in the hypoxia adaptation response [27,28,29]. This is consistent with our results in that both HIF-1α mRNA transcription in peripheral leukocytes as well as the HIF-1α protein levels in synovial tissue were significantly elevated in CIA mice. For the maintenance of energy homeostasis under hypoxia, the accumulation of HIF-1α promotes anaerobic glycolysis and leads to glycometabolic reprogramming in macrophages. Consequently, metabolic intermediates, such as pyruvate kinase M2 and succinate, which promote the expression and phosphorylation of STAT3, are accumulated [30,31,32]. Our results also showed increasing STAT3 and p-STAT3 levels in the synovial tissue of CIA mice.

STAT3 plays pivotal roles in inducing and maintaining the inflammatory microenvironment [33]. There is now considerable evidence that increasing phosphorylation of STAT3 aggravates NLRP3 expression [12,13]. As expected, NLRP3 expression in the synovial tissue of CIA mice was substantially elevated in the present study.

NLRP3 is an intracellular pattern-recognition receptor, which can be activated by a range of PAMPs and DAMPs [15]. The activation of NLRP3 leads to formation of the NLRP3 inflammasome, a cytoplasmic multimolecular platform. This inflammasome takes a pivotal role in IL-1β maturation and the subsequent inflammatory activation of macrophages [14]. Based on the above findings, we speculate that the HIF-1α/STAT3/NLRP3 axis directly connects the macrophages’ metabolic state with proinflammatory effector functions in the RA synovium, which might be a therapeutic target in RA treatment. Our results show that both the SDSS and MTX treatment significantly inhibited the HIF-1α/STAT3/NLRP3 pathway in CIA mice.

A previous study has shown that HIF-1α expression was evoked by LPS stimulation in vitro [34,35]. Similarly, HIF-1α expression was sharply increased by LPS stimulation in our study. Furthermore, LPS induced HIF-1α/STAT3/NLRP3 pathway activation, and a massive IL-1β secretion was inhibited by SDSS. These data illustrate that SDSS effectively inhibited the inflammatory activation in macrophages.

Combining outcomes in vitro and in vivo, we speculated that the HIF-1α/STAT3/NLRP3 pathway is the potential target for SDSS. Protein expression of the HIF-1α/STAT3/NLRP3 pathway, as well as downstream IL-1β secretion, was effectively suppressed by SDSS intervention. Furthermore, this effect was weakened by IOX4, a HIF prolyl hydroxylase inhibitor, which leads to the accumulation of HIF-1α in macrophages [36]. In the present study, stimulation of IOX4 resulted in HIF-1α accumulation, along with STAT3 phosphorylation and NLRP3 expression, as well as the secretion of IL-1β, in macrophages. As a result, the therapeutic effect of SDSS was attenuated.

However, there are some limitations in the present study. Firstly, although Western blotting was able to reflect protein expression in synovial tissues in our study, immunohistochemical experiments of macrophages infiltration, HIF-1α, STAT3 and NLRP3 expression in joints could show this more intuitively. Therefore, more work needs to be performed to confirm the effect of SDSS on the expression of these proteins in synovial tissue. Secondly, we proved that SDSS inhibited the expression of HIF-1α, but the potential mechanism is still unclear. HIF-1α is not only regulated by the oxygen-dependent pathway, but also by the NF-κB pathway [37]. A previous study has demonstrated that SDSS alleviated advanced glycation-end-product-mediated neuroinflammation in mice by inhibiting the NF-ĸB pathway [38]. Moreover, oxidative stress is involved in NF-ĸB pathway activation as well as in HIF-1α transcription [39]. It has been reported that DSS exhibited the most significant antioxidant property among the Danshen water extracts [40], and SDSS protected cells from oxidative damage via mitochondrial antioxidant defense systems [41]. Therefore, the antioxidant property of SDSS might play an important role in the inhibition of HIF-1α expression. Thirdly, as shown by Figure 6G, the SDSS-induced reduction of NLRP3 expression was not totally reversed by IOX4. This result implies that SDSS might inhibit NLRP3 expression through other pathways. Consequently, more experiments, such as gene interference in the HIF-1α/STAT3/NLRP3 pathway, are still required to clarify the role of SDSS in inflammatory activation in macrophages.

## 4. Methods

### 4.1. Animal Husbandry

DBA/1J mice were obtained from Beijing HFK Bioscience Co., Ltd. (Beijing, China). The license was SCXK Beijing 2020-0004. Mice were maintained in the SPF Laboratory Animal Center of Guangzhou University of Chinese Medicine. All of the animal experimental protocols were approved by Guangzhou University of Chinese Medicine Animal Ethics Committee (ethics approval number: 20210222006).

### 4.2. Collagen-Induced Arthritis (CIA) Induction

The process for CIA induction has been described previously [34]. In brief, bovine type II collagen (Chondrex, Woodinville, WA, USA, Cat#20021) and complete Freund’s adjuvant (Chondrex, Cat#7001) were fully emulsified. Then, 0.1 mL of the emulsion was injected into the tail root intradermally. The day of the primary injection was defined as day 0. A booster injection of 0.1 mL emulsion of bovine type II collagen and incomplete Freund’s adjuvant (Chondrex, Cat#7002) was intradermally injected into the back of mice on day 21. Meanwhile, normal saline was injected, in parallel, into mice of the control group.

### 4.3. Drug Administration

After the booster injection, mice with induced arthritis were randomly assigned into model group, SDSS group or MTX group (a positive control in the current study). SDSS (Selleck Chemicals, Houston, TX, USA, Cat#S2401) was given to the SDSS group by intragastric administration at 40 mg/kg every day. The doses of SDSS used in this study were determined by referring to previous studies [22,42].

MTX was given to the MTX group by intragastric administration at 2 mg/kg once every 3 days. This dosage is equivalent to the recommended dosages for humans undergoing RA treatment. MTX is commonly administered once per week in RA treatment. In CIA mice, MTX was administered once every 3 days in accordance with previous studies [43,44].

### 4.4. CIA Assessment

After the booster injection (on day 21), the hind-paw swelling of each mouse was measured weekly with digital caliper. In addition, a scoring system of 0–4 was used to determine clinical arthritis scores for each limb as previously described [45]. Body weight was recorded weekly.

### 4.5. Sample Collection

Blood samples from each mouse were harvested through the angular vein under isoflurane inhalation anesthesia; heparin was added to the samples to prevent coagulation. Samples were then centrifuged, at 3000 r for 15 min at 4 °C, to collect serum. Red cells were then dissolved with erythrocyte lysate, and the peripheral leukocytes were collected. In addition, limbs were harvested and dissected free of soft tissue. All the samples were kept at −80 °C or fixed in 4% paraformaldehyde until further assessment.

### 4.6. Splenic and Hepatosomatic Index Calculation

For splenic and hepatosomatic index calculation, the body weight of each mouse was recorded before blood sample collection. Then, the spleens and livers were isolated and weighed. Splenic index (%) = spleen weight/body weight × 100. Hepatosomatic index (%) = liver weight/body weight × 100.

### 4.7. Micro-Computed Tomography (Micro-CT) Scanning

Micro-CT scanning with Skyscan 1176 Micro-CT Imaging System (Skyscan, Belgium) was used to generate the two- and three-dimensional images of joints in the knee and ankle [34]. The scanning parameters settings were as follow: voltage, 80 kV; source current, 88 μA; and pixel size 4 μm. A data-viewer and CTvol software (Bruker micro-CT, Belgium) were used to generate the two- and three-dimensional images, respectively. A CT Analyzer program (Bruker micro-CT, Belgium) was used to measure the bone mineral density (BMD, mm^2^) and the bone volume/tissue volume (BV/TV, %) of the ankle and knee joints.

### 4.8. Hematoxylin-Eosin (HE) Staining

The left ankle joints were decalcified in EDTA for 8 weeks. After that, the samples were dehydrated by graded ethanol and were embedded with paraffin. Then, the samples were cut into 5 μm sagittal slices and stained with HE. Histological pictures were captured by a Leica microscope with a digital camera, and the histopathological score was determined according to a scoring system of 0–4, which has been reported previously [46].

### 4.9. Cell Culture

THP-1 cells were obtained from the American Type Culture Collection and maintained in RPMI-1640 (Biological industries, Cat#01-100-1ACS) supplemented with 0.05 mM β-mercaptoethanol (Solarbio, Cat#60-24-2) and 10% FBS (Biological industries, Cromwell, CT, USA, Cat#04-001-1ACS) at 37 °C in a humidified atmosphere of 5% CO_2_.

For adherent macrophages induction, THP-1 cells were maintained in complete medium supplemented with phorbol myristate acetate (100 ng/mL; Sigma, Livonia, MI, USA, Cat#P8139) for 24 h. Then, different concentrations of SDSS were used to pretreat these cells for 2 h before exposure to LPS (100 ng/mL; Sigma, Cat#L2630) stimulation for another 24 h. In addition, for stimulation with an HIF-1α agonist, cells were treated either with DMSO or IOX4 (Med Chem Express, Princeton, NJ, USA, Cat#HY-120110) dissolved in DMSO for 24 h.

### 4.10. Cell Viability Assay

The non-toxic concentration of SDSS in macrophages was selected using Cell Count Kit-8 (CCK8, Med Chem Express, Cat#HY-K0301) as previously described [34]. In brief, cells were planted in a 96-well plate and incubated with different concentrations of SDSS for 24 h. Then, cells were washed with phosphate buffered saline, and incubated, using RPMI-1640 media, with 10% CCK-8 solution for another 1–4 h. Finally, the absorbance of the supernatant was measured, and cell viability was calculated.

### 4.11. RNA Extraction and Real-Time PCR

Total RNA was isolated, using the Total RNA Kit II (OMEGA, Norcross, GA, USA, Cat#R6834-02) in RNase-free conditions from mouse peripheral leukocyte and treated THP-1-derived macrophages. Subsequently, cDNA was synthesized with the PrimeScript™ RT Master Mix kit (TaKaRa Bio, Kusatsu, Japan, Cat#RR036A). Then, real-time PCR was carried out using a 7500 Fast Real-Time PCR System (Carlsbad, CA, USA) with a TB Green^®^ Premix Ex Taq™ kit (TaKaRa Bio, Cat#RR420A). All of the primers were designed and synthesized by the Sangon Biotech (Shanghai, China) Co., Ltd. (Supplementary Table S1).

### 4.12. Western Blotting

Synovial tissues from the right forepaw joints and treated macrophages were lysed in RIPA buffer (Solarbio, Beijing, China, Cat#R0020) with phenylmethylsulfonyl fluoride (Solarbio, Cat#P0100) and protein phosphatase inhibitor (Solarbio, Cat#P1260). Protein samples were separated by SDS-PAGE. Separated proteins were transferred onto polyvinylidene difluoride membrane (Millipore, Burlington, MA, USA, Cat#IPVH00010). For specific protein detection, the following primary antibodies were used: rabbit anti-STAT3 (Cell Signaling Technology, Danvers MA, USA, Cat#9139S), rabbit anti-Phospho-STAT3 (Cell Signaling Technology, Cat#9145S), rabbit anti-NLRP3 (Invitrogen, Waltham, MA, USA, Cat# PA5-79740), mouse anti-HIF-1α (Santa Cruze, Dallas, TX, USA, Cat#13515), and rabbit anti-GAPDH (Abcam, Cambridge, UK, Cat# 8245). Lastly, the Western blotting bands were analyzed with Image J Software.

### 4.13. Cytokine Analysis

The analysis of IL-1β (CUSABIO, Houston, TX, USA, Cat#E08054m) and IL-6 (CUSABIO, Cat#E04639m) levels in the mouse serum and IL-1β (CUSABIO, Cat#E08053h) levels in cell supernatant were carried out using an ELISA kit.

### 4.14. Statistical Analysis

Data were given as mean values and standard deviation (SD). GraphPad Prism software (version 7.0) was used to draw graphs. Statistical analysis was performed with SPSS software (Version 17.0). Two-tailed unpaired Student’s *t* test was applied to data with normal distribution and homogeneous variance, whereas Dunnett’s T3 test was used for data with non-normal distribution. A *p* value of <0.05 was defined as significant.

## 5. Conclusions

This is the first in vivo and in vitro evidence that SDSS exerts its role in ameliorating arthritis through suppressing the HIF-1α/STAT3/NLRP3 pathway, therefore modulating the inflammatory activation in macrophages. This finding provides new insights into the pharmacological activities of SDSS and a potential therapeutic target for the treatment of RA as well.

## Data Availability

The data presented in this study are available upon request from the corresponding author.

## Supplementary Materials

The following supporting information can be downloaded at: https://www.mdpi.com/article/10.3390/molecules28041551/s1, Table S1: Oligonuceotide sequences for *Homo sapiens* and *Mus musculus* gene expansion.

## Sample Availability

Samples of the compounds are not available from the authors.

## Abbreviations

BMD	bone mineral density
BV/TV	bone volume/tissue volume
CCK8	cell count kit-8
CIA	collagen-induced arthritis
DAMPs	damage associated molecular patterns
DSS	Danshensu
HE	hematoxylin–eosin
HIF	hypoxia-inducible factor
IL	interleukin
Micro-CT	micro-computed tomography
MTX	methotrexate
NLRP	NOD-like receptor protein
PAMPs	pathogen-associated molecular patterns
PMA	phorbol myristate acetate
p-STAT3	phosphorylated STAT3
RA	rheumatoid arthritis
SD	standard deviation
SDSS	sodium Danshensu
STAT	signal transducer and activator of transcription
TNF	tumor necrosis factor
