# Supplementary material for: Suppression of Macrophage Activation by Sodium Danshensu via HIF-1α/STAT3/NLRP3 Pathway Ameliorated Collagen-Induced Arthritis in Mice
Source: Molecules. 2023 Feb 6;28(4):1551. doi: 10.3390/molecules28041551 (PMC9963181; doi:10.3390/molecules28041551)
Supplement: Supplementary file 1 [file molecules-28-01551-s001.zip › molecules-2057267-supplementary.pdf]

Supplementary Table S1. Oligonuceotide sequences for *Homo sapiens* and *Mus musculus* gene expansion.

Name	Primer sequence	
Homo sapiens		
	forward	AGCACAGTTACAGTATTCCAGCAGAC
HIF-1α	reverse	TTAATTCATCAGTGGTGGCAGTGGTAG
	forward	TGTGATGCTTCCCTGATTGTGACTG
STAT3	reverse	CTGGCAAGGAGTGGGTCTCTAGG
	forward	CTCGGTGACTTCGGAATCAGACTTC
NLRP3	reverse	GGGAATGGCTGGTGCTCAATACTG
Mus musculus		
	forward	GAATGAAGTGCACCCTAACAAG
HIF-1α	reverse	GAGGAATGGGTTCACAAATCAG
	forward	TGTCAGATCACATGGGCTAAAT
STAT3	reverse	GGTCGATGATATTGTCTAGCCA
	forward	GGAGGAAGAAGAAGAGAGGAGAGGAG
NLRP3	reverse	CTTGAGAAGAGACCACGGCAGAAG
